# Investigating the Biomarkers of the Sasang Constitution via Network Pharmacology Approach

**DOI:** 10.1155/2021/6665130

**Published:** 2021-04-13

**Authors:** Won-Yung Lee, Choong-Yeol Lee, Chang-Eop Kim, Ji-Hwan Kim

**Affiliations:** ^1^Department of Physiology, College of Korean Medicine, Gachon University, Seongnam, Republic of Korea; ^2^Department of Sasang Constitutional Medicine, College of Korean Medicine, Gachon University, Seongnam, Republic of Korea

## Abstract

Sasang constitutional (SC) medicine classifies people into Soeum (SE), Soyang (SY), Taeeum (TE), and Taeyang (TY) types based on psychological and physical traits. However, biomarkers of these types are still unclear. We aimed to identify biomarkers among the SC types using network pharmacology methods. Target genes associated with the SC types were identified by grouping herb targets that preserve and strengthen the requisite energy (*Bomyeongjiju*). The herb targets were obtained by constructing an herb-compound-target network. We identified 371, 185, 146, and 89 target genes and their unique biological processes related to SE, SY, TE, and TY types, respectively. While the targets of SE and SY types were the most similar among the target pairs of the SC types, those of TY type overlapped with only a few other SC-type targets. Moreover, SE, SY, TE, and TY were related to “diseases of the digestive system,” “diseases of the nervous system,” “endocrine, nutritional, and metabolic diseases,” and “congenital malformations, deformations, and chromosomal abnormalities,” respectively. We successfully identified the target genes, biological processes, and diseases related to each SC type. We also demonstrated that a drug-centric approach using network pharmacology analysis provides a deeper understanding of the concept of Sasang constitutional medicine at a phenotypic level.

## 1. Introduction

The Sasang constitutional medicine (SCM) is a unique form of personalized medicine within traditional Korean medicine, which uses a constitutional typology for medical purposes. Unlike traditional Chinese medicine, which classifies the organs into five organ systems, the SCM theory classifies human physiological and pathological phenomena as four organ systems, which are paired: the lung, spleen, liver, and kidney. The liver-lung pair and spleen-kidney pair exhibit a functional symmetrical relationship similar to a seesaw. A hyperactive kidney and hypoactive spleen are traits unique to the Soeum (SE) type, and *vice versa* for the Soyang (SY) type. A hyperactive liver and hypoactive lung are applicable to the Taeeum (TE) type, and *vice versa* for the Taeyang (TY) type. The combination of their functional deviations represents the characteristics of the four Sasang constitutional (SC) types [1]. To identify those “inherent” characteristics, SCM experts categorize patients as one of the four SC types by comprehensively considering appearance, body shape, personality, and usual health status [2, 3]. Using the diagnostic information of a patient's SC type, an SCM expert can identify the risk factors for certain SC-type-specific symptoms and provide a patient with tailored treatment. Several studies have reported that chronic diseases such as hypertension, obesity, and metabolic syndrome exhibit an SC-type-specific susceptibility [4–7]. Thus, although there may be controversy over the accuracy of the model, which classifies individual differences into four types according to the philosophical theory of a seesaw-like relationship, SCM can provide valuable experiences and helpful insights for improvement in personalized medicine, especially, in predicting prognosis and providing tailored interventions.

A prerequisite step for securing scientific evidence for SCM and expanding its application in personalized medicine is to identify biomarkers of each SC type. Previous studies, employing genome-wide association study (GWAS) and metabolomics study, have revealed genetic variations and metabolite-level biomarkers in healthy people [8–12]. However, the diagnosis of an SC type in these studies ultimately relied on the SCM doctors or questionnaires and was thus subject to bias due to the doctor's subjective clinical experience or low sensitivity of the questionnaire [13]. Therefore, there is still a pressing need for another approach that elucidates the biomarkers of SC types without bias.

**Figure 1:**
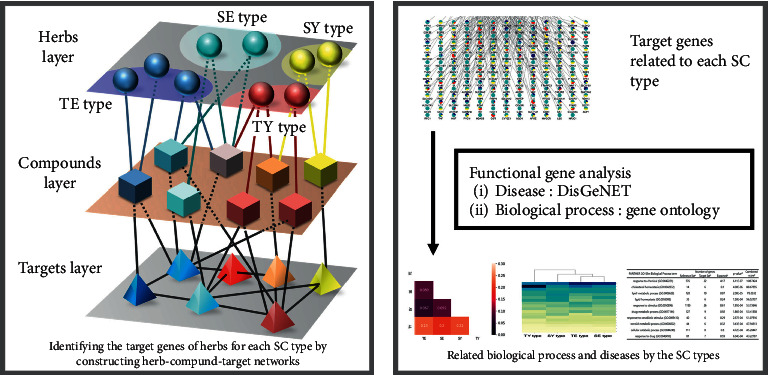
Overview of the study process. (a) Network construction. The SC-type-related networks were constructed by conducting a network pharmacology analysis on the herbs that preserve and strengthen the requisite energy in each SC type and integrating the obtained SC-type herb-compound-target interaction information. (b) Functional gene analysis. The functions and diseases for each SC type were identified by performing functional gene analyses on the target genes according to the SC type. SE: Soeum, SY: Soyang, TE: Taeeum, and TY: Taeyang.

On the basis of the SCM theory, we devised an alternative strategy to identify the biomarkers of the SC type. The energy (Qi) derived from the hypoactive organ is referred to as “the requisite energy (*Bomyeongjiju*, a life-sustaining source of power)” [14]. When this requisite energy is depleted, the equilibrium states of the visceral function of the SC types are disturbed and manifest as SC-type-specific symptoms or diseases. To alleviate such pathologies, corresponding medicinal herbs that preserve and strengthen the requisite energy for each SC type are prescribed. Consequently, the analysis of the mechanisms of medicinal herbs related to the requisite energy will help identifying the biomarkers associated with the inherent paired organs of each SC type and facilitate a deeper understanding of the SC types. We explored the mechanisms of these medicinal herbs using network pharmacology methods, which have emerged as promising tools for elucidating and analyzing the mechanisms of drugs at the system level [15–17]. The target genes of each SC type were identified by constructing the SC-type-related networks, and their related functions and diseases were analyzed and visualized (Figure 1). Given that these medicinal herbs are administered to treat a pathological condition specific to an SC type, the proposed approach, which we call it as “drug-centric approach,” can provide clues for understanding biomarkers of the SC type at a phenotypic level.

## 2. Materials and Methods

### 2.1. Identification of Herbs

The list of herbs that strengthen and preserve the requisite energy (*Bomyeongjiju*) was obtained from *Dongmuyugo*, a classic Korean medical text written by *Je-Ma Lee* [18]. Among the books written by *Je-Ma Lee*, this is the only book that explicitly classifies medicinal herbs that enhance and preserve the energy needed for the SE, SY, and TE types. Another book written by *Je-Ma Lee*, *Donguisusebowon* (Longevity & Life Preservation in Eastern Medicine), describes more abundant herbs that are prescribed for various symptoms and diseases of each SC type; nevertheless, it does not provide explicit links between herbs and the requisite energy [19]. However, even in *Dongmuyugo*, the herbs that conserve and strengthen the requisite energy for the TY type are not defined. Therefore, these herbs were inevitably defined as the common herbs in *Ohgaphijangchuck-tang* and *Meehudeungsikjang-tang*, the only two prescriptions for the TY type specified in *Donguisusebowon* [20]. The Latin names of herbs were retrieved from the KIOM herbarium (https://boncho.kiom.re.kr/herbarium/codex.php) and the Korean Herbal Pharmacopoeia. The KIOM herbarium is a publicly accessible website that provides curated information, such as the origin and Latin names of herbal medicines recorded in the Korean, Chinese, Japanese, Taiwanese, and North Korean pharmacopoeias. The above curation process was conducted after consultation with Korean doctors specialized in herbology or SCM.

### 2.2. Identification of Target Genes

The information of the target genes for each SC-type-related herb was obtained by constructing an herb-compound-target network using TCM-Mesh (http://mesh.tcm.microbioinformatics.org) [21]. TCM-Mesh is an online tool that provides a network pharmacology analysis of herbal medicines in a high-throughput manner by integrating information from various sources. For obtaining the information on compound-target interactions, the confidence for the interaction was set to the default value suggested by TCM-Mesh (combined score > 700). The score was calculated using a model that predicts the drug-target interactions based on a random forest model [22]. We limited the maximum number of target genes for a compound to 10 to avoid a biased result due to small sets of compounds with an immoderate number of target genes.

### 2.3. Comparison and Visualization of Target Genes

InteractiVenn (http://www.interactivenn.net) was employed to construct Venn diagrams between the target sets of the SC types [23]. This tool offers a clean interface for Venn diagram construction and enables the analysis of set unions while preserving the shape of the diagram. The target genes for each SC type were hierarchically clustered using the unweighted pair group method with arithmetic mean clustering algorithm with cosine distance.

### 2.4. Functional Annotation and Analysis

The functional annotation and gene ontology overrepresentation analyses were performed using the online analytical tool PANTHER (Protein ANalysis THrough Evolutionary Relationships; http://www.pantherdb.org, v.14.0) [24]. PANTHER is widely used as a comprehensive resource for gene function classification and genome-wide data analysis. Fisher's exact tests with the Benjamini–Hochberg false discovery rate correction were employed to determine the significance of gene ontology (GO) terms in the biological process category of the *Homo sapiens* genome. The *Z*-score of each GO term was computed for the deviation from an expected rank using a modification to Fisher's exact tests. Finally, the combined scores were calculated by multiplying the *Z*-scores and the logarithms of *p* values (this combined score was notably different from the combined score in TCM-Mesh).

The involvement on diseases of each SC type was analyzed according to the procedure of a previously described method [25]. In brief, the gene-disease interactions were retrieved from the DisGeNET (https://www.disgenet.org/, v.6.0) [26]. The DisGeNET provides a score for manually curated gene-disease interactions between 0 and 1, taking into account the number of sources supporting the association and the reliability of each of them. The top three scored diseases associated with each target gene were obtained and classified based on the “International Statistical Classification of Diseases and Related Health Problems, 10th Revision (ICD-10): Version 2016” [27].

## 3. Results

### 3.1. Herb List Related to the Requisite Energy

To investigate the biological correlates of the SC types, we first identified the list of herbs that preserve and strengthen the requisite energy for each SC type. This list of herbs was retrieved from *Dongmuyugo* or defined using herbal formulae (see *Materials and Methods*). Among the herb list, *Pruni Arillus* (*Aengdoyuk*, the TY-type-related herb), which is not included in TCM-Mesh, was excluded from this study. Finally, a total of 28 herbs were included in the study (Table 1).

### 3.2. Target Genes Related to SC Type

**Figure 2:**
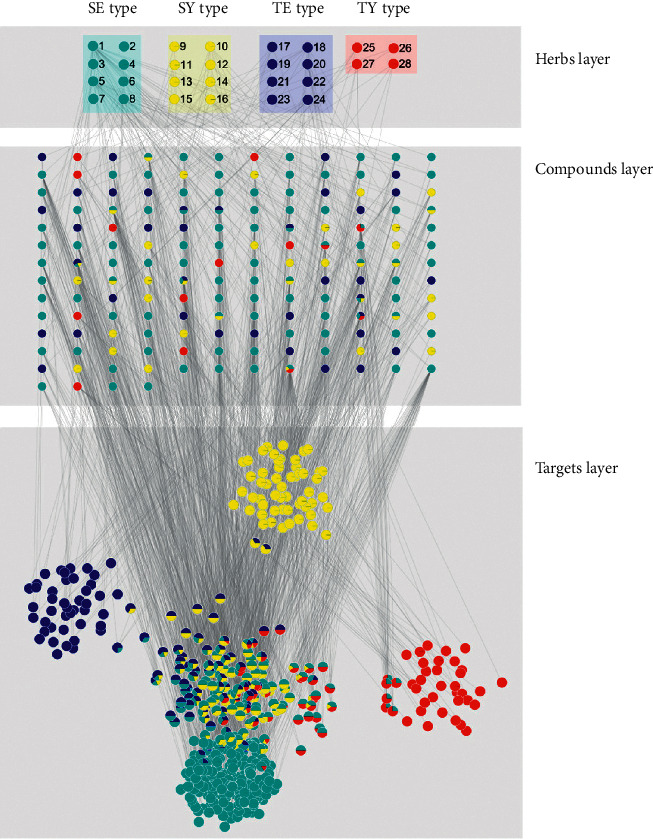
Representative herb-compound-target networks for herbs related to the requisite energy. The circles in each layer represent herbs, compounds, or targets. The pie charts in each node denote proportions related to the SC types (Blue, cyan, yellow, and red colors represent TE, SE, SY, and TY types, respectively.). The herb no. (see Table 1) and gene symbols are attached to the nodes in the herbs layer and targets layer. SE: Soeum, SY: Soyang, TE: Taeeum, and TY: Taeyang.

**Figure 3:**
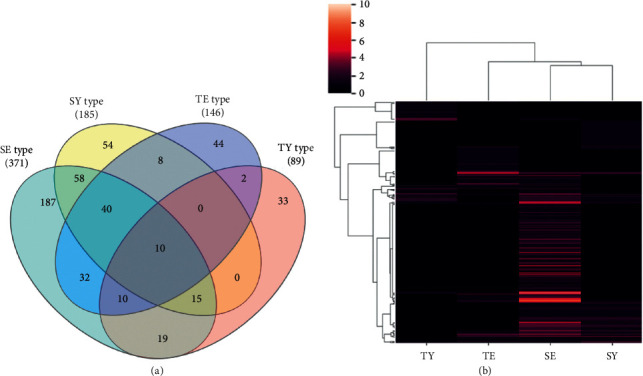
Comparison among the related targets of Sasang constitutional (SC) types. (a) Venn diagram of the targets related to SC type. The number indicates the targets that were shared between or unique to each SC type. (b) Clustered heatmap of the targets related to SC types. Each line represents a specific target, and the color of the line indicates the number of herbal compounds interacting with each target. The data are displayed in a heatmap and clustered with respect to both targets and SC types. SE: Soeum, SY: Soyang, TE: Taeeum, and TY: Taeyang.

To identify the target genes related to the SC types, we constructed an herb-compound-target network for each herb using TCM-Mesh (Figure 2 and Supplementary Table 1). The network consisted of three types of nodes (herbs, compounds, and targets) and two types of edges (between herbs and compounds and between compounds and targets). SC-type-related genes were retrieved by collecting those target genes of each SC type. We identified 371, 185, 146, and 89 related target genes of the SE, SY, TE, and TY types, respectively. We observed that the SE type and SY type shared the greatest number of target genes (*n* = 58) among the SC type pairs and so were grouped at the first level in the hierarchical clustering (Figure 3). This indicated that the target genes of the SE-SY pair were most similar among the target pairs of the SC type. On the other hand, the TY type shared only a few target genes with other SC types (2, 19, and 0 for the TY-TE, TY-SE, and TY-SY pairs, respectively) and clustered at the last level, indicating that the target genes of the TY type were relatively distinct from those of other SC types.

Since we had limited the maximum number of target genes for a compound to 10, we tested whether this constraint biased the similarity relationship among the SC types. Therefore, we clustered the target genes related to the SC types by changing the threshold of the maximum target gene number (*n* = 10, 50, 100, 500, and no limit) and compared the order of clustering between them. The results showed that the hierarchy of clusters between the SC types did not change with varying thresholds, indicating that the similarity relationship among the SC types did not arise from the maximum number of target genes (Supplementary Figure 1).

Additionally, we tested whether the proportion of the target genes exclusively belonging to a specific SC type was higher than the values in the null distribution. The values of the null distribution were obtained by randomly shuffling the compound-target interactions and then repeatedly calculating the proportion of target genes belonging to a specific SC type. The results showed that the proportion of exclusive target genes in the original data was much higher than the chance level (*p* value <0.001), indicating that the group of target genes showed a unique pattern for each SC type.

### 3.3. Biological Correlates Related to Functional Gene Analysis

**Figure 4:**
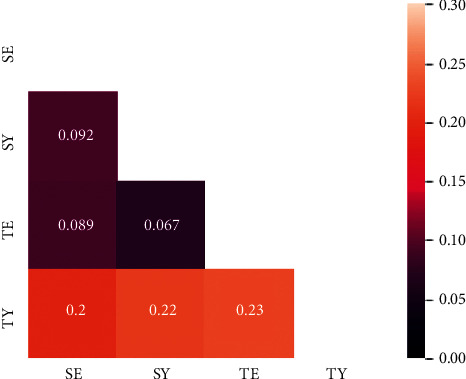
Distance matrix of the SC types. The color bar indicates the Euclidian distance between the SC types in terms of their related biological processes. SE: Soeum, SY: Soyang, TE: Taeeum, and TY: Taeyang.

We annotated and analyzed the related functions of the target genes associated with the SC types. This analysis allows us to identify the biological processes associated with the target genes of each SC type. Every target gene of the compound-target network was assigned to first-level categories of biological processes using the “GO-Slim biological process” in the PANTHER classification system (v. 14.0) [24]. Among these categories, cellular processes, metabolic processes, and biological regulation were highly associated with the target genes of the SC types (Supplementary Table 2). In particular, more than half of the target genes of the TY type were found to be involved in metabolic processes. Simultaneously, we also calculated the Euclidean distance for the proportion of the categories of biological processes for each SC-type pair (Figure 4). The SY-TE pair presented the lowest distance among all constitution pairs, followed by SE-TE and SE-SY pairs (0.067, 0.089, and 0.092, respectively). On the other hand, all TY-type-related pairs showed the greatest distance from the other pairings (0.2, 0.22, and 0.23 for TY-SE, TY-SY, and TY-TE pairs, respectively), thus indicating that the function of the TY type was very different from the other SC types.

Next, we conducted an overrepresentation test to investigate the specific biological processes significantly associated with each SC type using PANTHER (v.14.0). The results showed that 74, 86, 43, and 60 biological process terms were significantly associated to the SE, SY, TE, and TY types, respectively (adjusted *p* < 0.05, Tables 2–5, and Supplementary Tables 3–6). Among the top 10 terms, ranked by the combined score, the drug metabolic process (GO:0017144) and response to chemical (GO:0042221) terms were commonly associated with the SE, SY, and TE types. On the other hand, each SC type showed association with distinct terms in the biological process category. The SE type was significantly related to various metabolic processes (GO:0008152) (cellular metabolic process, fatty acid metabolic process, cofactor metabolic process, carbohydrate metabolic process, cellular lipid metabolic process, ammonium ion metabolic process, nitrogen compound metabolic process; Table 3), whereas the SY type was significantly associated with the positive regulation of lymphocyte proliferation (GO:0050671), leukocyte proliferation (GO:0070665), and mononuclear cell proliferation (GO:0032946) (Table 4). While the TE type was significantly associated with lipid homeostasis (GO:0006629)–related and lipid metabolism (GO:0055088)-related terms (cholesterol homeostasis, lipid metabolic process, lipid homeostasis, steroid metabolic process; Table 2), the TY type was significantly related to ATP-associated metabolic processes (GO:0046034) (mitochondrial electron transport, cytochrome c to oxygen, and ATP metabolic process; Table 5).

**Figure 5:**
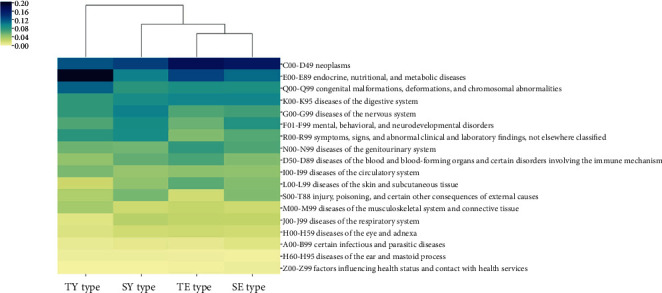
Related disease categories by Sasang constitutional (SC) type. The color of each cell denotes the proportion of diseases associated with each SC type. SE: Soeum, SY: Soyang, TE: Taeeum, and TY: Taeyang.

To characterize the diseases in each SC type, we analyzed and compared the association of the three top-scoring disease categories with each SC type using DisGeNET (Figure 5). The “neoplasms” showed a high frequency in most of the groups, a result consistent with that of the previous research that identified diseases related to medical herbs [25]. On the other hand, each SC type showed differences in the annotated disease classifications; “endocrine, nutritional, and metabolic diseases” showed the highest frequency (20.0%) in the TE type, and “diseases of the digestive system” showed the third highest frequency (9.4%) in the SE type. “Diseases of the nervous system” and “mental, behavioral, and neurodevelopmental disorders” showed the second and seventh highest prevalence, respectively (9.8% and 7.8%), in the SY type, and “congenital malformations, deformations, and chromosomal abnormalities” showed the third highest frequency (11.3%) in the TY type.

## 4. Discussion

Through this study, we have addressed a crucial issue in SCM by demonstrating that a drug-centric approach can successfully uncover novel biomarkers for each SC type with related biological functions and diseases. The drug-centric approach often refers to a drug discovery strategy that links known drugs to new targets and their related indications [28]. However, in this study, it refers to the alternative strategy of identifying biomarkers associated with the SC type, as established by the SCM theory with inherent paired organs and the requisite energy, by using the modern approach of network pharmacology for analyzing medicinal herbs that are classified according to the same SCM theory. The permutation test supports that the group of target genes between SC types is clearly distinguished compared with the random distribution, and this unique pattern is reflected in the results for biological processes and diseases.

It is noteworthy that our results of the SC-type-related genes differ from those of previous studies, which addressed the characteristics of genetic background for each SC type. Kim et al. found that polymorphisms of some genes, such as FTO, MC4R, and interleukin-1*α* and *β*, were associated with SC type [8–10], which was not corroborated by our results. Such inconsistency may have been derived from the research hypotheses and the employed experimental techniques. Above-mentioned previous studies have identified genetic variations using GWAS based on the hypothesis that there are inherited characteristics associated with specific genes that are related to phenotypic traits of each SC type. On the other hand, we focused on the targets of herbs and their associated biological processes and diseases based on the hypothesis that medicinal herbs prescribed to patients are the key to understanding the pathophysiological traits of the SC type [18]. Since SCM is actually based on the clinical practice using specific herbs for each SC type, our drug-centric approach can provide a further explanation about the pathophysiological traits of the SC types in the clinical setting.

Our cluster analysis revealed that the target genes between the SE type and SY type were most similar among the target gene list of the SC-type pairs (Figure 3). This result may seem controversial because the SE and SY types exhibit opposite activities with respect to the kidney and spleen groups [1]. One possible explanation for this contradictory result is that the medicinal herbs assigned to the SE and SY types act on the overlapped target gene through the opposite mode of action. Since current network pharmacology methods only predict simple interactions without the mode of action, further research is needed for identifying biomarkers of the SC type that exhibit upregulation and downregulation. However, our results on the Euclidian distance of the related biological process among the SC types indicated that the SY type and TE type were the closest pair in the biological process category. The two types share more similarity than the SE or TY type in that they show a cold pattern in exterior disease and a heat pattern in internal disease, while SE shows the reverse pattern, and TY does not show a distinct heat or cold pattern [20]. Our results additionally showed that the biological processes, as well as target genes, of the TY type are different from those of the other SC types. Further research on this aspect is needed.

The functional gene analysis revealed the related biological process or diseases for each SC type. Although the underlying mechanisms of the SC-type-specific diseases were not completely revealed, the results from the analysis provide clues to identify the underlying pathophysiology of the diseases for each SC type. For example, our results showed that the TE type is closely related to lipid homeostasis–related and lipid metabolism–related biological process terms and also highly related to the “endocrine, nutritional, and metabolic disease.” Indeed, recent studies have shown that lipid homeostasis and lipid metabolism are closely associated with obesity, diabetes, and metabolic syndrome [29–31], and the prevalence of these diseases was found to be highest in the TE type [5, 32, 33]. Our results also identified that the SE type was closely associated with metabolic processes and the “disease of the digestive system.” Similarly, recent researches revealed that the irritable bowel syndrome, a disease highly prevalent in the SE type [4], is positively related to the expression of genes involved in metabolic processes [34]. These consistent results prove that the techniques employed in our study are reliable and can be used to generate new hypotheses for future studies. Specifically, our results can help reveal the pathophysiological traits of the SY and the TY types, which have not yet been studied extensively.

One major advantage of the drug-centric approach is that the SC types cannot be falsely labeled. In the previous studies [8–12], the SC types were classified by the SCM doctors, after considering the patient's morphological and psychological traits. However, its diagnostic procedure can be biased by the doctor's subjective clinical experience, which means that the relative importance of traits contributing to SC-type diagnosis can vary from doctor to doctor. This situation often leads to the diagnosis of different SC types for the same person. One study reported that the accordance rates of SC-type diagnosis between SCM experts were 52.5–68.4%, according to SC types [35]. Another advantage is that the drug-centric approach can identify biomarkers of rare constitutions that may be difficult to study in a population. For instance, the Korea Constitutional Multicenter Bank (KCMB), the largest biobank of SCM, revealed that the constitutional distributions for TY type were nonzero but below 0.1%, deeming it too rare to include in a population-based study [36]. To the best of our knowledge, we first identified biomarkers of the TY type, leveraging prior knowledge from the classic Korean medical text.

There are some limitations to our study as well. First, our results are based on the hypothesis of Je-Ma Lee, so our findings require additional validation using preclinical and clinical studies. Second, our results cannot determine whether the target genes or biological functions are activated or inhibited in each SC type. An interesting objective for future research will be identifying which target genes or biological processes are upregulated or downregulated, to shed more light on the seesaw balance between the organ groups of specific pairs. Third, our scope of analysis has focused on the herbs that are explicitly related to the requisite energy, except certain frequently prescribed medicinal herbs such as *Aconiti Lateralis, Radix Preparata*, *Gypsum Fibrosum*, and *Puerariae Radix* for the SE, SY, and TY types, respectively. In spite of these limitations, our findings provide a deeper understanding of the concept of SCM at the biological level.

## 5. Conclusion

In this study, we identified biomarkers of SC type by employing network pharmacology. The target genes, biological processes, and diseases showed unique patterns among the SC types, and they were consistent with the results of previous studies. Furthermore, we showed that the proposed strategy can provide reliable hypothesis for SCM, which broadens the application of personalized medicine by bridging the gap between SCM and modern medicine.

## Figures and Tables

**Table 1:** List of herbs related to the requisite energy.

No.	Sasang constitutional type	Local name	Latin name
1	SE type	Insam (renshen)	*Ginseng Radix*
2		Baekchul (baizhu)	*Atractylodis Rhizoma Alba*
3		Jagamcho (zhigancao)	*Glycyrrhizae Radix et Rhizoma Praeparata cum Melle*
4		Danggui (danggui)	*Angelicae Gigantis Radix*
5		Chungung (chuanxiong)	*Cnidii Rhizoma*
6		Gwangye (guangui)	*Cortex Cinnamomi*
7		Jinpi (chenpi)	*Citri Unshius Pericarpium*
8		Baekjakyak (baishaoyao)	*Paeoniae Radix*
9	SY type	Sugjihwang (shudihuang)	*Rehmanniae Radix Preparata*
10		Sansuyu (shanzhuyu)	*Corni Fructus*
11		Bokryeong (fufing)	*Poria Sclerotium*
12		Jimo (zhimu)	*Anemarrhenae Rhizoma*
13		Taegsa (zexie)	*Alismatis Rhizoma*
14		Moktong (mutong)	*Akebiae Caulis*
15		Mokdanpi (mudanpi)	*Moutan Radicis Cortex*
16		Huangbaek (huangbai)	*Phellodendri Cortex*
17	TE type	Maengmundong (maimendong)	*Liriopis seu Ophiopogonis Tuber*
18		Omija (wuweizi)	*Schisandrae Fructus*
19		Sadang (shatang)	*Saccharum*
20		Sanyak (shanyao)	*Dioscoreae Rhizoma*
21		Gilgyeong (jiegeng)	*Platycodonis Radix*
22		Uwang (niuhuang)	*Bostaurus Linne var. domesticus Gmelin*
23		Seokchangpo (shichangpu)	*Acori Graminei Rhizoma*
24		Hunaggeum (huangqin)	*Scutellariae Radix*
25	TY type^#^	Ogapi (wejiapi)	*Acanthopanacis Cortex*
26		Mogwa (mugua)	*Chaenomelis Fructus*
27		Podogeun (putaogen)	*Radix Vitis Romanetii*
28		Nogeun (lugen)	*Phragmitis Rhizoma*

SE: Soeum, SY: Soyang, TE: Taeeum, and TY: Taeyang. # The herbs for the TY type were selected from herbal formulae prescribed for the TY type (see *Materials and Methods* for detail).

**Table 2:** Gene ontology terms of the biological process category associated with Soeum type.

PANTHER GO-Slim biological process term	Number of genes			*p* value^d^	Combined score^e^
	Reference list^a^	Target list^b^	Expected^c^		
Response to xenobiotic stimulus (GO:0009410)	40	12	0.37	5.19*E* − 12	205.5949
Drug metabolic process (GO:0017144)	127	21	1.19	5.60*E* − 12	342.0589
Response to drug (GO:0042493)	81	16	0.76	8.09*E* − 09	252.1907
Terpenoid metabolic process (GO:0006721)	11	5	0.1	9.17*E* − 09	63.41361
Isoprenoid metabolic process (GO:0006720)	14	5	0.13	4.93*E* − 08	57.00586
Diterpenoid metabolic process (GO:0016101)	10	4	0.09	2.56*E* − 07	41.92858
Positive regulation of leukocyte proliferation (GO:0070665)	3	2	0.03	3.39*E* − 07	16.30587
Regulation of smooth muscle contraction (GO:0006940)	3	2	0.03	3.56*E* − 07	16.26849
Positive regulation of mononuclear cell proliferation (GO:0032946)	3	2	0.03	1.14*E* − 06	16.21936
Positive regulation of lymphocyte proliferation (GO:0050671)	3	2	0.03	2.53*E* − 06	16.18301

^a^Number of genes in the reference list that map to this PANTHER classification category. ^b^Number of genes in the target gene list that map to this PANTHER classification category. ^c^The expected value is the number of genes that can be expected in the target gene list for this PANTHER category based on the reference list. ^d^*p* values are determined by binomial statistics with Benjamini–Hochberg corrections. ^e^Combined score is computed by taking the log of the *p* value and multiplying that by the *Z*-score of the deviation from the expected rank.

**Table 3:** Gene ontology terms of the biological process category associated with Soyang type.

PANTHER GO-Slim biological process term	Number of genes			*p* value^d^	Combined score^e^
	Reference list^a^	Target list^b^	Expected^c^		
Cellular metabolic process (GO:0044237)	1744	84	32.73	7.02*E* − 16	230.1149
Drug metabolic process (GO:0017144)	127	23	2.38	5.71*E* − 13	226.2333
Fatty acid metabolic process (GO:0006631)	138	20	2.59	2.50*E* − 11	144.3277
Response to chemical (GO:0042221)	576	39	10.81	9.88*E* − 10	141.426
Response to xenobiotic stimulus (GO:0009410)	40	12	0.75	4.88*E* − 08	123.8619
Cofactor metabolic process (GO:0051186)	159	19	2.98	1.32*E* − 07	107.5996
Cellular lipid metabolic process (GO:0044255)	294	25	5.52	3.47*E* − 07	103.8276
Carbohydrate metabolic process (GO:0005975)	185	20	3.47	5.99*E* − 07	103.5943
Ammonium ion metabolic process (GO:0097164)	34	10	0.64	1.28*E* − 06	92.36633
Nitrogen compound metabolic process (GO:0006807)	38	10	0.71	1.41*E* − 06	85.06531

^a^Number of genes in the reference list that map to this PANTHER classification category. ^b^Number of genes in the target gene list that map to this PANTHER classification category. ^c^The expected value is the number of genes that can be expected in the target gene list for this PANTHER category based on the reference list. ^d^*p* values are determined by binomial statistics with Benjamini–Hochberg corrections. ^e^Combined score is computed by taking the log of the *p* value and multiplying that by the *Z*-score of the deviation from the expected rank.

**Table 4:** Gene ontology terms of the biological process category associated with Taeeum type.

PANTHER GO-Slim biological process term	Number of genes			p-value^d^	Combined score^e^
	Reference list^a^	Target list^b^	Expected^c^
Response to chemical (GO:0042221)	576	22	4.17	6.11*E* − 07	108.7424
Cholesterol homeostasis (GO:0042632)	14	6	0.1	4.00*E* − 06	88.67855
Lipid metabolic process (GO:0006629)	120	10	0.87	2.08*E* − 05	73.0932
Lipid homeostasis (GO:0055088)	33	6	0.24	1.50*E* − 04	56.52157
Response to stimulus (GO:0050896)	1190	26	8.61	1.95*E* − 04	53.73646
Drug metabolic process (GO:0017144)	127	9	0.92	1.86*E* − 04	53.41338
Response to xenobiotic stimulus (GO:0009410)	40	6	0.29	2.37*E* − 04	51.07916
Steroid metabolic process (GO:0008202)	44	6	0.32	3.43*E* − 04	47.94313
Cellular catabolic process (GO:0044248)	111	8	0.8	4.72*E* − 04	45.28447
Response to drug (GO:0042493)	81	7	0.59	6.04*E* − 04	43.22787

^a^Number of genes in the reference list that map to this PANTHER classification category. ^b^Number of genes in the target gene list that map to this PANTHER classification category. ^c^The expected value is the number of genes that can be expected in the target gene list for this PANTHER category based on the reference list. ^d^*p* values are determined by binomial statistics with Benjamini–Hochberg corrections. ^e^Combined score is computed by taking the log of the *p*-value and multiplying that by the Z-score of the deviation from the expected rank.

**Table 5:** Gene ontology terms of the biological process category associated with Taeyang type.

PANTHER GO-Slim biological process term	Number of genes			*p* value^d^	Combined score^e^
	Reference list^a^	Target list^b^	Expected^c^		
Inorganic anion transport (GO:0015698)	44	9	0.19	2.13*E* − 09	164.4011
Mitochondrial electron transport, cytochrome c to oxygen (GO:0006123)	185	13	0.79	2.33*E* − 09	159.3545
Nitric oxide biosynthetic process (GO:0006809)	54	9	0.23	3.18*E* − 09	152.3056
Sensory perception of sweet taste (GO:0050916)	55	9	0.24	3.66*E* − 09	152.0322
Arginine catabolic process (GO:0006527)	66	9	0.28	1.01*E* − 08	138.1824
Negative regulation of blood pressure (GO:0045776)	10	6	0.04	1.11*E* − 08	137.2376
Regulation of lyase activity (GO:0051339)	42	8	0.18	1.12*E* − 08	137.1961
Positive regulation of phosphate metabolic process (GO:0045937)	45	8	0.19	1.21*E* − 08	134.0532
Secondary metabolite biosynthetic process (GO:0044550)	45	8	0.19	1.36*E* − 08	133.5836
ATP synthesis coupled electron transport (GO:0042773)	4072	45	17.45	1.48*E* − 08	131.5255

^a^Number of genes in the reference list that map to this PANTHER classification category. ^b^Number of genes in the target gene list that map to this PANTHER classification category. ^c^The expected value is the number of genes that can be expected in the target gene list for this PANTHER category based on the reference list. ^d^*p* values are determined by binomial statistics with Benjamini–Hochberg corrections. ^e^Combined score is computed by taking the log of the *p* value and multiplying that by the *Z*-score of the deviation from the expected rank.

## Data Availability

The data used to support the findings of this study are included within the supplementary information files.

## Supplementary Materials

Supplementary Materials Figure S1: cluster map of the targets associated with Sasang constitution types by changing the threshold of the number of targets (*n* = 10, 50, 100, 500, and no limit) of a compound. Supplementary Table 1: target genes related to each Sasang constitutional type. Supplementary Table 2: biological processes related to the targets of each Sasang constitutional type. Supplementary Table 3: gene ontology terms of the biological process category associated with Soeumin type. Supplementary Table 4: gene ontology terms of the biological process category associated with Soyangin type. Supplementary Table 5: gene ontology terms of the biological process category associated with Taeeumin type. Supplementary Table 6: gene ontology terms of the biological process category associated with Taeyangin type.
