# Supplementary material for: Investigating the Biomarkers of the Sasang Constitution via Network Pharmacology Approach
Source: Evid Based Complement Alternat Med. 2021 Apr 13;2021:6665130. doi: 10.1155/2021/6665130 (PMC8060121; doi:10.1155/2021/6665130)
Supplement: Supplementary Materials — Figure S1: cluster map of the targets associated with Sasang constitution types by changing the threshold of the number of targets (n = 10, 50, 100, 500, and no limit) of a compound. Supplementary Table 1: target genes related to each Sasang constitutional type. Supplementary Table 2: biological processes related to the targets of each Sasang constitutional type. Supplementary Table 3: gene ontology terms of the biological process category associated with Soeumin type. Supplementary Table 4: gene ontology terms of the biological process category associated with Soyangin type. Supplementary Table 5: gene ontology terms of the biological process category associated with Taeeumin type. Supplementary Table 6: gene ontology terms of the biological process category associated with Taeyangin type. [file 6665130.f1.pdf]

Fig S1. Clustermap of the targets associated with Sasang constitution types by changing the threshold of the number of targets (n= 10, 50, 100, 500 and no limit) of a compound. SE: Soeumin, SY: Soyangin, TE: Taeeumin, and TY: Taeyangin.

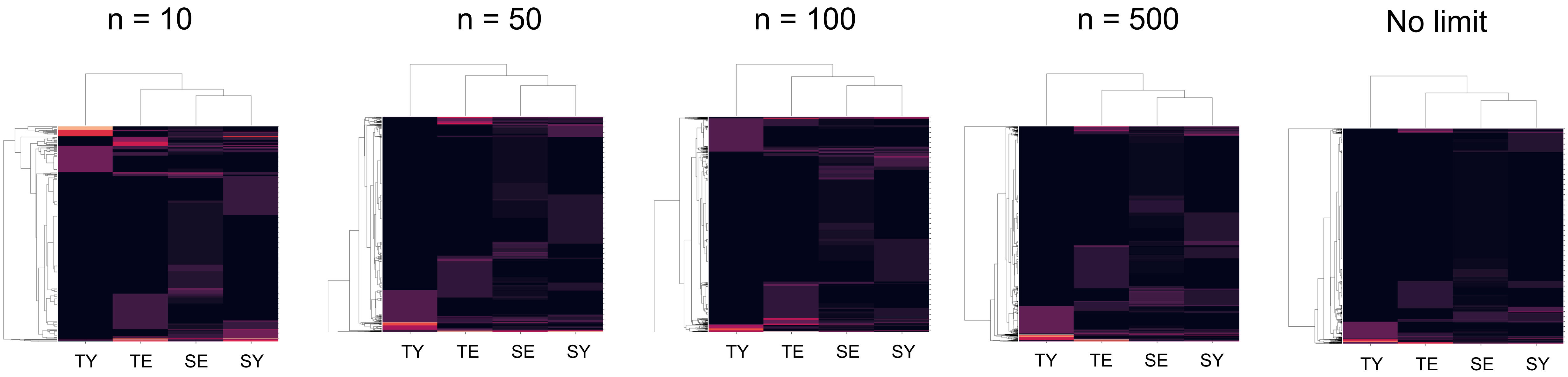

Supplementary table 1. Target genes related to each Sasang constitutional type

Soeumin t	Soyangin	Taeeumin	Taeyangin type
CFTR	CASP3	NR1I2	MTHFR
CASP3	PTGS2	ABCG5	GSR
GCG	CHAT	ABCG8	BLVRB
CYP1A1	CYP3A4	CASP3	FLAD1
AHRR	ABCG5	CYP1A2	ACP5
NR1I2	ABCG8	PTGS2	PYGL
MAPK1	ALDH2	CYP3A4	ENPP1
CYP1A2	AKT1	MMP9	NOS3
AR	PPARA	ABCA1	UGT1A1
AKT1	MMP9	MMP2	TP53
BID	BCHE	SLCO1B1	INS
AKR1B1	CPA6	NR1H2	SI
PPARA	PARP1	LST1	SLC22A1
CES2	ALDH3A1	CYP1B1	SCD
TRPA1	CYP2A6	ABCC1	TPK1
MAPK3	TP53	VEGFA	SLC22A2
CES1	SLC22A1	AKT1	SLC22A5
FABP4	CASP8	SREBF2	SLC19A2
ALB	ABCA1	CYP1A1	TKT
SULT1A1	ACHE	AHRR	NMT1
F2	SREBF2	SULT1A1	SLC19A3
FABP3	CYP2E1	SLC28A3	CFTR
HSD11B2	ICAM1	SLC28A2	SLC2A1
PTGS2	CHKB	DHCR24	RPL3
CHAT	CKB	ACAT1	CYP3A4
SRC	ZNF32	SOAT1	AKT1
TRAPPC3	ADRA2A	APOB	HBA1
CPT2	SLC44A1	BAX	HBA2
CD1D	PLD2	MAPK8	CYP2B6
MMP9	SLC5A7	UGT1A1	PNP
CD1A	CHKA	SULT1A2	CCND1
UGT1A10	SLC44A2	SULT4A1	UCK2
UGT1A7	HBA1	HMOX1	AR
CYP2B6	HBA2	TIMP2	AKR1B1
TNF	EIF2AK3	BCHE	UGT1A6
BCHE	HBB	PARP1	TAS1R3
PPT1	HBD	TP53	LCT
FABP1	GCG	CASP8	UGT1A4
EDN1	CYP1A1	HBA1	CD4
UGT1A8	AHRR	HBA2	TAS1R2
CYP1B1	CYP1A2	HBB	CYP4F3

FOS	SULT1A1	HBD	CYP4F2
CPA6	F2	GCG	UGT1A10
SAT2	UGT1A10	F2	UGT1A7
ABCB1	UGT1A7	CYP2B6	UGT1A8
INS	CYP2B6	TNF	TYR
CSF2	TNF	INS	TYRL
PCSK1	EDN1	SLC29A2	RNASE1
AKR1C2	UGT1A8	PNP	SRC
SAT1	FOS	CCND1	HK3
PARP1	ABCB1	GSTP1	GANC
ALDH3A1	INS	MAOB	KHK
CYP2A6	CYP2A13	MAOA	HK2
SRM	SCD	CDA	ALDOC
CYP2A13	ALDH3A2	PYGM	GALM
SMS	TPK1	SLC28A1	MDH2
TP53	SLC28A3	UPP1	FHIT
SCD	SLC28A2	UCK2	GLA
SLC22A1	SLC29A2	UCK1	MT-CO2
SLC22A11	CAT	UCKL1	ME3
FASN	PNP	APOE	CES3
CASP8	SLC22A2	NT5C	CENPE
ABCA1	CCND1	HMGCR	LEP
ALDH7A1	TYR	DRD1	AS3MT
ODC1	SLC22A5	TAAR1	COX7C
ABCG5	SLC19A2	DRD2	INSR
AHR	TKT	REN	PDE5A
ALDH3A2	NMT1	DRD3	COX6B1
LMNA	SLC19A3	MAPK1	ME1
ACHE	GSTP1	AR	ACO2
SREBF2	TYRL	AKR1B1	BTK
AMD1	MAPK14	TRPA1	DYT17
HSD11B1	MAOB	MAPK3	MT-CO1
CYP2E1	GSTM1	PCSK1	ACLY
ABCG8	ADRA2C	AKR1C2	VCL
ICAM1	CASP9	LMNA	PLEKHA1
MTHFR	MAOA	NT5C2	CS
FFAR1	ADA	NQO1	SEPT5
NT5C2	MMP2	HGF	RBKS
FAS	NFE2L2	CXCL8	HS3ST3A1
PAOX	DHCR24	IL6	GAA
GJA1	RBP5	HIF1A	SLC2A2
CHKB	CDA	SI	COX6A2
CKB	PECR	SLC29A1	COX7A1

ZNF32	SLCO1B1	COMT	COX5A
TPK1	AOC3	HPRT1	MT-CO3
CD1B	AOC2	F9	SNORD83B
SMOX	CTGF	SLC6A3	NOS2
SLC28A3	NR1H2	NT5E	COX5B
ANPEP	ACAT1	CCL2	
SLC5A6	LST1	ADCY2	
MTHFS	GSTA1	SLC29A3	
NQO1	PYGM	UGT1A6	
ADRA2A	SLC28A1	APOA1	
EGFR	FFAR2	VKORC1	
HGF	CDKN1B	CDK2	
CYP3A4	DHRS3	TAS1R3	
SLC28A2	RDH11	FFAR4	
SLC29A2	UPP1	LCT	
UGT1A9	RBP4	UGT1A4	
GSK3B	RBP2	CD4	
OXTR	ESR1	TAS1R2	
SLC6A4	ACP1	BDNF	
AHCY	LRAT	BGLAP	
PMCH	APRT	CCL17	
HK3	MRI1	CDKN1A	
LSS	KNG1	EGF	
DAO	MTAP	LCAT	
BHLHE40	UCK2	CYP11A1	
ALDH2	UCK1	CYP4F3	
SLC44A1	RBP1	PROS1	
PLD2	HEBP1	TBK1	
MAPK8	RDH8	SCN8A	
CXCL8	SOAT1	KCNK10	
GNG2	GNRH1	ALOXE3	
HTT	PLA2G7	CBR1	
FPGS	UCKL1	ATF1	
IL6	APOB	SCN3A	
CAT	APOE	ALLC	
FMO3	RDH12	PTH	
PNP	ABCC11	CYP7A1	
GPR132	RNASE1	CDX2	
SLC22A2	NT5C	KRAS	
RGS19	RDH10	ALDH8A1	
CCND1	HMGCR	GC	
SMO	ADRA2B	CAMK2A	
NR3C1	ARL1	CALR	

TYR	DRD1	FOXP3
SLC22A5	PNMT	SLC35A2
SLC19A2	ADH1C	HRAS
GAST	NNMT	CYP19A1
HIF1A	SELE	PTGS1
SI	IMPA1	CFLAR
TKT	SLC2A13	LPL
SLC5A7	ATD	HPGDS
MPO	CHRNA4	GGCX
RFC1	BST1	IL17A
HDAC6	ADH1B	STK11
DFNB38	BAX	CYP2C19
FLOT1	TAAR1	MTTP
GLRX	SLC47A2	ALOX12
DHFR	TH	DRD4
NMT1	PTGIS	CYP4F2
RYR2	NAMPT	TRPM8
SLC19A3	PTGIR	MGP
GSTP1	SLC5A3	NPC1
CA2	CALCA	
ABCC1	ADRA1A	
TYRL	GAMT	
CHKA	SLC8A1	
MAPK14	SLC22A3	
MAOB	DRD2	
TYMS	ADRA1D	
GSTM1	RIPK4	
GSR	MIOX	
EDN3	UGT2B7	
SLC44A2	TYRO3	
ADRA2C	REN	
UGT1A1	SIRT1	
CRKL	ATM	
SLC19A1	UXS1	
CCKBR	HTR1D	
CASP9	IMPAD1	
MAOA	TNFSF11	
CD1C	NMNAT1	
PTGR2	CD38	
ADA	INPP1	
MMP2	SLC5A11	
ATIC	HTR1B	
PRKACA	DGAT2	

MMP7	SIRT5
PC	IMPA2
FAR2	AKR1B10
NFE2L2	TNKS2
DHCR24	SLC47A1
DHDH	CYP2D6
GANC	FDFT1
GLYAT	HTR2A
AKR1C3	DDC
TAAR5	CYP2C9
RBP5	JUN
BLVRB	DLG4
FLAD1	CDIPT
MAN1A1	DRD3
PANK4	PPARG
FAR1	
PON1	
SLC29A1	
ALOX15	
CDA	
KHK	
PTER	
PECR	
SLCO1B1	
ACP5	
AOC3	
AGPS	
ANGPT2	
RPS6KA3	
TOP2A	
COMT	
VWF	
PCCA	
VNN2	
HSP90AA1	
HPRT1	
AOC2	
CA1	
CTGF	
SLC27A6	
DOHH	
ACACA	
ALDH1A3	

NR1H2
ACAT1
LST1
HK2
GSTA1
STRA13
EGR1
F9
PYGM
AGO2
SLC28A1
PYGL
NPY1R
ALDH3B2
SLC6A3
FFAR2
CDKN1B
KIF11
ADK
XRCC1
MAN1C1
DHRS3
AQP1
RDH11
MET
CYP2F1
HSD17B10
NT5E
UPP1
SULT1A2
HSPA4
PRDX6
SLC27A4
ACSL1
LSM2
RBP4
CREB1
PTPN2
ALDOC
ADORA1
EDEM2
ACSM2B
RBP2

CCL2
GLTP
ESR1
GYG1
VNN1
MAN1B1
PANK1
PLA2G1B
MAN1A2
ACSL4
ACP1
LRAT
SLC27A1
BTD
PPCDC
ENPP1
MITF
EIF5A2
ADCY2
PTK2
APRT
MRI1
ADORA2B
KNG1
MTAP
EDEM3
EBF1
UCK2
SLC29A3
ANGPTL4
UGT1A6
UCK1
KLK3
RBP1
PLA2G2A
VEGFA
PANK2
APOA1
RAB9A
HLCS
ACSM1
UGT3A1
PRDX5

FADS1
CYB5A
CRYAA
HEBP1
DECR1
FADS2
FZD8
RDH8
GALM
SOAT1
MDH2
PPCS
FAAH
NFKBIA
PANK3
VKORC1
HBA1
FHIT
AOC1
CDK2
GNRH1
PLA2G7
AZI2
PDYN
OR6A2
LTC4S
ANGPTL2
KIAA0101
MGAT2
UCKL1
NPY5R
ALDH9A1
TAS1R3
APOB
NOS3
GLA
APOE
RDH12
P2RX1
GNPAT
UST
EDEM1
FFAR4

P2RY1
LCT
ABCC11
ALDH1B1
ADORA2A
RNASE1
HBA2
NT5C
NPY2R
DGAT1
CCNA2
P2RY2
RAD52
ADORA3
UGT1A4
HAS2
CD4
SLCO2B1
CA14
PARG
TAS1R2
MCEE
RDH10
CCK
HMGCR
BDNF
ADRA2B
PPP1R1B
LSM1

Supplementary table 2. Biological processes related to the targets of each Sasang constitutional type.

(unit : proportion)

Category name	Taeeumin t	Soeumin t	Soyangin t	Taeyangin t
cellular process (GO:0009987)	0.382	0.376	0.434	0.4
metabolic process (GO:0008152)	0.296	0.312	0.301	0.5
biological regulation (GO:0065007)	0.217	0.173	0.219	0.144
response to stimulus (GO:0050896)	0.171	0.117	0.163	0.133
localization (GO:0051179)	0.132	0.091	0.117	0.122
multicellular organismal process (GO:00	0.099	0.069	0.066	0.067
immune system process (GO:0002376)	0.046	0.033	0.031	0.011
cell proliferation (GO:0008283)	0.02	0.015	0.01	0.011
biological adhesion (GO:0022610)	0.013	0.018	0.02	0
developmental process (GO:0032502)	0	0.005	0	0.011
reproduction (GO:0000003)	0.007	0.005	0	0
cellular component organization or bio	0	0.003	0	0
biological phase (GO:0044848)	0	0.003	0	0

Targets were assigned to first-level categories of biological processes using “Panther GO-slim Biological Process”.

Supplementary table 3. Gene ontology terms of the biological process category associated with Soeumin type.

PANTHER GO-Slim Biological Process	no	Homo sap	Client Tex	Client Tex	Client Tex	Client Tex	Client Tex	Client Tex	z-score	combined score
cellular metabolic process (GO:0044237)	2	1744	84	32.73	+	2.57	3.12E-15	5.60E-12	6.889472	230.1149
drug metabolic process (GO:0017144)	1	127	23	2.38	+	9.65	5.77E-15	5.19E-12	6.90028	226.2333
fatty acid metabolic process (GO:0006631)	3	138	20	2.59	+	7.72	1.35E-11	8.09E-09	5.766572	144.3277
response to chemical (GO:0042221)	4	576	39	10.81	+	3.61	2.04E-11	9.17E-09	5.745407	141.426
response to xenobiotic stimulus (GO:0009410)	5	40	12	0.75	+	15.99	1.37E-10	4.93E-08	5.453817	123.8619
cofactor metabolic process (GO:0051186)	6	159	19	2.98	+	6.37	8.55E-10	2.56E-07	5.153258	107.5996
cellular lipid metabolic process (GO:0044255)	8	294	25	5.52	+	4.53	1.39E-09	3.56E-07	5.091096	103.8276
carbohydrate metabolic process (GO:0005975)	7	185	20	3.47	+	5.76	1.51E-09	3.39E-07	5.100364	103.5943
ammonium ion metabolic process (GO:0097164)	9	34	10	0.64	+	15.67	5.70E-09	1.14E-06	4.865791	92.36633
nitrogen compound metabolic process (GO:0006	10	38	10	0.71	+	14.02	1.41E-08	2.53E-06	4.705697	85.06531
metabolic process (GO:0008152)	11	4072	123	76.41	+	1.61	3.93E-08	6.42E-06	4.512063	76.93989
cellular aromatic compound metabolic process (G	12	77	12	1.44	+	8.3	8.55E-08	1.28E-05	4.363496	71.01481
monocarboxylic acid metabolic process (GO:0032	13	50	10	0.94	+	10.66	1.33E-07	1.84E-05	4.283471	67.81984
lipid metabolic process (GO:0006629)	14	120	14	2.25	+	6.22	1.90E-07	2.43E-05	4.221205	65.32839
cellular nitrogen compound metabolic process (G	15	68	11	1.28	+	8.62	2.12E-07	2.54E-05	4.211216	64.7124
cellular response to chemical stimulus (GO:00708	16	208	18	3.9	+	4.61	2.27E-07	2.55E-05	4.210328	64.41093
carboxylic acid metabolic process (GO:0019752)	17	55	10	1.03	+	9.69	2.91E-07	3.08E-05	4.167469	62.72017
organic substance biosynthetic process (GO:1901	19	265	20	4.97	+	4.02	3.77E-07	3.76E-05	4.121753	60.96494
monosaccharide metabolic process (GO:0005996)	18	90	12	1.69	+	7.11	3.92E-07	3.71E-05	4.124835	60.84959
steroid metabolic process (GO:0008202)		44	9	0.83	+	10.9	4.83E-07	4.33E-05	4.089127	59.46919
coenzyme metabolic process (GO:0006732)	21	76	11	1.43	+	7.71	5.76E-07	4.93E-05	4.058921	58.31516
oxoacid metabolic process (GO:0043436)	22	67	10	1.26	+	7.95	1.47E-06	1.20E-04	3.846126	51.65443
organonitrogen compound metabolic process (G	23	191	16	3.58	+	4.46	1.59E-06	1.24E-04	3.83808	51.24519
Unclassified (UNCLASSIFIED)	24	10588	151	198.69	-	0.76	2.04E-06	1.53E-04	3.786149	49.60825
organic acid metabolic process (GO:0006082)	25	73	10	1.37	+	7.3	2.95E-06	2.12E-04	3.70427	47.16909
lipid localization (GO:0010876)	26	92	11	1.73	+	6.37	3.15E-06	2.18E-04	3.697189	46.83639
response to stimulus (GO:0050896)	27	1190	46	22.33	+	2.06	5.57E-06	3.71E-04	3.559897	43.06805
lipid transport (GO:0006869)	28	83	10	1.56	+	6.42	8.32E-06	5.34E-04	3.463095	40.5073
organic substance metabolic process (GO:007170	29	3338	97	62.64	+	1.55	1.12E-05	6.91E-04	3.393125	38.68026
cellular amide metabolic process (GO:0043603)	30	23	6	0.43	+	13.9	1.25E-05	7.47E-04	3.371721	38.066
cellular catabolic process (GO:0044248)	31	111	11	2.08	+	5.28	1.62E-05	9.41E-04	3.307597	36.48445
nucleobase-containing small molecule metabolic	32	157	13	2.95	+	4.41	1.69E-05	9.48E-04	3.305521	36.32172
small molecule metabolic process (GO:0044281)	33	221	15	4.15	+	3.62	3.48E-05	1.89E-03	3.106994	31.89607
response to drug (GO:0042493)	34	81	9	1.52	+	5.92	4.22E-05	2.23E-03	3.057757	30.80107
catabolic process (GO:0009056)	35	262	16	4.92	+	3.25	6.35E-05	3.26E-03	2.942095	28.43379
organic substance transport (GO:0071702)	36	112	10	2.1	+	4.76	8.86E-05	4.42E-03	2.84652	26.56195
acyl-CoA metabolic process (GO:0006637)	37	35	6	0.66	+	9.14	9.80E-05	4.76E-03	2.822842	26.05637
cholesterol transport (GO:0030301)	38	12	4	0.23	+	17.76	1.73E-04	8.18E-03	2.644548	22.90766
nucleoside phosphate metabolic process (GO:000	39	126	10	2.36	+	4.23	2.18E-04	1.00E-02	2.575829	21.71686
cellular process (GO:0009987)	40	6070	148	113.91	+	1.3	2.63E-04	1.18E-02	2.51807	20.75735
cholesterol homeostasis (GO:0042632)	41	14	4	0.26	+	15.23	2.83E-04	1.24E-02	2.500552	20.42967

response to antibiotic (GO:0046677)	42	5	3	0.09	+	31.97	3.24E-04	1.39E-02	2.459837	19.76422
folic acid-containing compound metabolic proces	43	16	4	0.3	+	13.32	4.35E-04	1.82E-02	2.361524	18.27858
response to toxic substance (GO:0009636)	44	91	8	1.71	+	4.68	4.91E-04	2.00E-02	2.326348	17.7246
organic substance catabolic process (GO:1901575	45	196	12	3.68	+	3.26	5.03E-04	2.01E-02	2.324476	17.65421
organelle organization (GO:0006996)	46	771	3	14.47	-	0.21	5.56E-04	2.13E-02	2.302624	17.25757
nucleotide metabolic process (GO:0009117)	47	117	9	2.2	+	4.1	5.52E-04	2.15E-02	2.299087	17.24766
macromolecule localization (GO:0033036)	48	172	11	3.23	+	3.41	6.08E-04	2.27E-02	2.278446	16.87266
cellular macromolecule localization (GO:0070727)	49	857	4	16.08	-	0.25	6.55E-04	2.35E-02	2.265207	16.60595
cellular protein localization (GO:0034613)	51	853	4	16.01	-	0.25	6.51E-04	2.39E-02	2.258733	16.57233
long-chain fatty acid transport (GO:0015909)	50	7	3	0.13	+	22.84	6.76E-04	2.38E-02	2.260343	16.49896
cellular modified amino acid metabolic process ((	52	54	6	1.01	+	5.92	8.13E-04	2.81E-02	2.195887	15.62325
nucleotide biosynthetic process (GO:0009165)	53	76	7	1.43	+	4.91	8.51E-04	2.89E-02	2.184848	15.4449
vesicle-mediated transport (GO:0016192)	54	643	2	12.07	-	0.17	8.85E-04	2.94E-02	2.178081	15.31174
positive regulation of adaptive immune response	55	21	4	0.39	+	10.15	1.06E-03	3.33E-02	2.128447	14.57877
pigment biosynthetic process (GO:0046148)	56	21	4	0.39	+	10.15	1.06E-03	3.39E-02	2.121259	14.52954
neuromuscular synaptic transmission (GO:000727	57	21	4	0.39	+	10.15	1.06E-03	3.45E-02	2.114179	14.48104
positive regulation of immune response (GO:005(	58	22	4	0.41	+	9.69	1.23E-03	3.56E-02	2.101467	14.08139
regulation of neurotransmitter levels (GO:000150	60	22	4	0.41	+	9.69	1.23E-03	3.62E-02	2.094674	14.03587
secondary metabolic process (GO:0019748)	61	9	3	0.17	+	17.76	1.21E-03	3.67E-02	2.089086	14.03267
pigment metabolic process (GO:0042440)	63	22	4	0.41	+	9.69	1.23E-03	3.68E-02	2.087976	13.99099
folic acid-containing compound biosynthetic proc	64	9	3	0.17	+	17.76	1.21E-03	3.73E-02	2.082465	13.9882
organic acid biosynthetic process (GO:0016053)	59	82	7	1.54	+	4.55	1.29E-03	3.61E-02	2.095799	13.94359
carboxylic acid biosynthetic process (GO:0046394	62	82	7	1.54	+	4.55	1.29E-03	3.67E-02	2.089086	13.89892
purine nucleobase biosynthetic process (GO:0009	65	10	3	0.19	+	15.99	1.55E-03	4.27E-02	2.026633	13.1113
polysaccharide metabolic process (GO:0005976)	66	62	6	1.16	+	5.16	1.57E-03	4.28E-02	2.025656	13.07901
cellular glucan metabolic process (GO:0006073)	71	25	4	0.47	+	8.53	1.87E-03	4.94E-02	1.965123	12.34454
cellular component organization (GO:0016043)	67	1723	16	32.33	-	0.49	1.99E-03	4.76E-02	1.980922	12.32058
purine nucleobase transport (GO:0006863)	68	2	2	0.04	+	53.29	1.98E-03	4.81E-02	1.976484	12.30294
nucleobase transport (GO:0015851)	69	2	2	0.04	+	53.29	1.98E-03	4.87E-02	1.971209	12.2701
purine nucleoside monophosphate biosynthetic p	73	11	3	0.21	+	14.53	1.94E-03	4.98E-02	1.961678	12.25081
nucleoside transport (GO:0015858)	70	2	2	0.04	+	53.29	1.98E-03	4.94E-02	1.965123	12.23222
positive regulation of immune system process (G	72	26	4	0.49	+	8.2	2.13E-03	4.96E-02	1.963398	12.0781

Supplementary table 4. Gene ontology terms of the biological process category associated with Soyangin type.

PANTHER GO-Slim Biological Process	no	Homo sap	Client Tex	Client Tex	Client Tex	Client Tex	Client Tex	Client Tex	z-score	combined score
drug metabolic process (GO:0017144)	1	127	21	1.19	+	17.71	3.91E-19	7.02E-16	8.070171	342.0589
response to drug (GO:0042493)	2	81	16	0.76	+	21.16	6.36E-16	5.71E-13	7.207234	252.1907
response to xenobiotic stimulus (GO:0009410)	3	40	12	0.37	+	32.14	4.17E-14	2.50E-11	6.673367	205.5949
response to chemical (GO:0042221)	4	576	28	5.38	+	5.21	2.20E-12	9.88E-10	6.111337	164.0439
cellular catabolic process (GO:0044248)	5	111	13	1.04	+	12.55	1.36E-10	4.88E-08	5.455628	123.943
cellular metabolic process (GO:0044237)	6	1744	45	16.28	+	2.76	4.42E-10	1.32E-07	5.276047	113.6445
G-protein coupled receptor signaling pathway, coup	7	136	13	1.27	+	10.24	1.35E-09	3.47E-07	5.095948	104.0754
cellular response to chemical stimulus (GO:0070887	8	208	15	1.94	+	7.73	2.67E-09	5.99E-07	4.991539	98.53891
adenylate cyclase-activating G-protein coupled rece	9	77	10	0.72	+	13.91	7.85E-09	1.28E-06	4.842834	90.38062
activation of adenylate cyclase activity (GO:0007190	10	77	10	0.72	+	13.91	7.85E-09	1.41E-06	4.823586	90.02139
cellular aromatic compound metabolic process (GO:	11	77	10	0.72	+	13.91	7.85E-09	1.57E-06	4.802113	89.62065
response to stimulus (GO:0050896)	12	1190	32	11.11	+	2.88	9.17E-08	1.37E-05	4.348615	70.46819
adenylate cyclase-modulating G-protein coupled re	13	112	10	1.05	+	9.56	2.04E-07	2.62E-05	4.204206	64.76641
cellular lipid metabolic process (GO:0044255)	15	294	15	2.74	+	5.47	2.04E-07	2.81E-05	4.188342	64.52202
regulation of adenylate cyclase activity (GO:004576	14	113	10	1.05	+	9.48	2.21E-07	2.64E-05	4.202486	64.40353
terpenoid metabolic process (GO:0006721)	16	11	5	0.1	+	48.69	2.59E-07	2.90E-05	4.18118	63.41361
small molecule metabolic process (GO:0044281)	18	221	13	2.06	+	6.3	2.94E-07	3.11E-05	4.165258	62.64418
regulation of cAMP-mediated signaling (GO:004394	17	118	10	1.1	+	9.08	3.21E-07	3.03E-05	4.1712	62.36705
regulation of cyclase activity (GO:0031279)	19	118	10	1.1	+	9.08	3.21E-07	3.20E-05	4.158746	62.18084
isoprenoid metabolic process (GO:0006720)	20	14	5	0.13	+	38.26	6.73E-07	6.04E-05	4.011243	57.00586
small molecule catabolic process (GO:0044282)	21	49	7	0.46	+	15.3	8.22E-07	7.03E-05	3.975268	55.69957
G-protein coupled receptor signaling pathway (GO:(	22	309	14	2.88	+	4.85	2.01E-06	1.64E-04	3.768852	49.43745
Unclassified (UNCLASSIFIED)	23	10588	66	98.84	-	0.67	2.67E-06	2.08E-04	3.709097	47.60044
neuromuscular synaptic transmission (GO:0007274)	24	21	5	0.2	+	25.51	3.61E-06	2.70E-04	3.642503	45.64713
response to toxic substance (GO:0009636)	25	91	8	0.85	+	9.42	3.87E-06	2.78E-04	3.634982	45.30008
regulation of catalytic activity (GO:0050790)	26	203	11	1.9	+	5.8	5.21E-06	3.60E-04	3.567794	43.40196
diterpenoid metabolic process (GO:0016101)	27	10	4	0.09	+	42.85	6.61E-06	4.39E-04	3.515456	41.92858
fatty acid metabolic process (GO:0006631)	28	138	9	1.29	+	6.99	9.45E-06	6.06E-04	3.428915	39.67081
cholesterol transport (GO:0030301)	29	12	4	0.11	+	35.71	1.18E-05	7.33E-04	3.376929	38.3194
organic substance transport (GO:0071702)	30	112	8	1.05	+	7.65	1.61E-05	9.64E-04	3.300828	36.43022
cellular process (GO:0009987)	31	6070	85	56.66	+	1.5	2.38E-05	1.38E-03	3.198802	34.05388
lipid localization (GO:0010876)	32	92	7	0.86	+	8.15	3.78E-05	2.12E-03	3.072885	31.29181
regulation of molecular function (GO:0065009)	33	360	13	3.36	+	3.87	4.69E-05	2.48E-03	3.025771	30.15935
drug transport (GO:0015893)	34	38	5	0.35	+	14.1	4.65E-05	2.53E-03	3.01973	30.125
catabolic process (GO:0009056)	35	262	11	2.45	+	4.5	5.01E-05	2.57E-03	3.014976	29.85275
organic substance biosynthetic process (GO:190157	36	265	11	2.47	+	4.45	5.53E-05	2.76E-03	2.993276	29.3423
alcohol metabolic process (GO:0006066)	37	68	6	0.63	+	9.45	6.37E-05	3.09E-03	2.95864	28.58438
monocarboxylic acid metabolic process (GO:003278	38	50	5	0.47	+	10.71	1.54E-04	7.26E-03	2.684677	23.56759
cofactor metabolic process (GO:0051186)	39	159	8	1.48	+	5.39	1.68E-04	7.72E-03	2.664077	23.15495
regulation of signal transduction (GO:0009966)	40	359	12	3.35	+	3.58	1.85E-04	7.90E-03	2.656314	22.83143
regulation of cell communication (GO:0010646)	41	359	12	3.35	+	3.58	1.85E-04	8.09E-03	2.64829	22.76247

regulation of cellular process (GO:0050794)	42	1126	24	10.51	+	2.28	1.83E-04	8.22E-03	2.642897	22.74484
carboxylic acid metabolic process (GO:0019752)	43	55	5	0.51	+	9.74	2.32E-04	9.71E-03	2.585989	21.64156
macromolecule localization (GO:0033036)	44	172	8	1.61	+	4.98	2.79E-04	1.14E-02	2.530192	20.70785
aromatic compound biosynthetic process (GO:0019	45	12	3	0.11	+	26.78	3.27E-04	1.30E-02	2.483769	19.93362
anterograde trans-synaptic signaling (GO:0098916)	48	330	11	3.08	+	3.57	3.50E-04	1.34E-02	2.472958	19.67875
synaptic signaling (GO:0099536)	46	331	11	3.09	+	3.56	3.59E-04	1.32E-02	2.478327	19.65856
trans-synaptic signaling (GO:0099537)	49	331	11	3.09	+	3.56	3.59E-04	1.34E-02	2.472958	19.61597
chemical synaptic transmission (GO:0007268)	50	330	11	3.08	+	3.57	3.50E-04	1.37E-02	2.465035	19.6157
metabolic process (GO:0008152)	47	4072	59	38.01	+	1.55	3.71E-04	1.33E-02	2.475634	19.55579
organophosphate metabolic process (GO:0019637)	51	137	7	1.28	+	5.47	3.93E-04	1.38E-02	2.462428	19.30962
ammonium ion metabolic process (GO:0097164)	52	34	4	0.32	+	12.6	4.10E-04	1.41E-02	2.454706	19.14512
reactive oxygen species metabolic process (GO:007	53	35	4	0.33	+	12.24	4.53E-04	1.51E-02	2.429972	18.70986
organic cyclic compound biosynthetic process (GO:	57	35	4	0.33	+	12.24	4.53E-04	1.54E-02	2.422833	18.65489
cell death (GO:0008219)	54	342	11	3.19	+	3.45	4.68E-04	1.53E-02	2.425199	18.5941
cholesterol homeostasis (GO:0042632)	56	14	3	0.13	+	22.95	4.81E-04	1.54E-02	2.422833	18.50958
purine nucleobase transport (GO:0006863)	55	2	2	0.02	+	> 100	5.04E-04	1.54E-02	2.422833	18.39641
cell-cell signaling (GO:0007267)	60	523	14	4.88	+	2.87	4.97E-04	1.57E-02	2.415816	18.37692
nucleobase transport (GO:0015851)	58	2	2	0.02	+	> 100	5.04E-04	1.56E-02	2.418142	18.36079
organonitrogen compound metabolic process (GO:1	59	191	8	1.78	+	4.49	5.46E-04	1.56E-02	2.418142	18.16724
cell communication (GO:0007154)	64	527	14	4.92	+	2.85	5.35E-04	1.60E-02	2.408916	18.14695
oxoacid metabolic process (GO:0043436)	62	67	5	0.63	+	7.99	5.45E-04	1.58E-02	2.413503	18.13681
regulation of intracellular signal transduction (GO:1	63	293	10	2.74	+	3.66	5.43E-04	1.60E-02	2.408916	18.11119
organic substance metabolic process (GO:0071704)	61	3338	50	31.16	+	1.6	5.59E-04	1.57E-02	2.415816	18.09292
nitrogen compound metabolic process (GO:000680	65	38	4	0.35	+	11.28	6.03E-04	1.67E-02	2.393247	17.74256
organic acid metabolic process (GO:0006082)	67	73	5	0.68	+	7.34	7.86E-04	2.14E-02	2.300852	16.44776
positive regulation of leukocyte proliferation (GO:00	66	3	2	0.03	+	71.41	8.36E-04	2.14E-02	2.300852	16.30587
regulation of smooth muscle contraction (GO:00069	69	3	2	0.03	+	71.41	8.36E-04	2.17E-02	2.295578	16.26849
nucleobase-containing small molecule metabolic pr	68	157	7	1.47	+	4.78	8.52E-04	2.16E-02	2.297329	16.23735
positive regulation of mononuclear cell proliferatior	70	3	2	0.03	+	71.41	8.36E-04	2.21E-02	2.288645	16.21936
positive regulation of lymphocyte proliferation (GO:	71	3	2	0.03	+	71.41	8.36E-04	2.24E-02	2.283516	16.18301
organophosphate catabolic process (GO:0046434)	72	18	3	0.17	+	17.85	9.16E-04	2.29E-02	2.275099	15.91544
apoptotic process (GO:0006915)	73	321	10	3	+	3.34	1.07E-03	2.62E-02	2.223234	15.20714
programmed cell death (GO:0012501)	74	327	10	3.05	+	3.28	1.22E-03	2.96E-02	2.175402	14.59456
lipid transport (GO:0006869)	76	83	5	0.77	+	6.45	1.35E-03	3.24E-02	2.139441	14.13668
pigment biosynthetic process (GO:0046148)	75	21	3	0.2	+	15.3	1.37E-03	3.23E-02	2.140678	14.11337
pigment metabolic process (GO:0042440)	77	22	3	0.21	+	14.61	1.54E-03	3.60E-02	2.096927	13.57965
regulation of leukocyte proliferation (GO:0070663)	78	5	2	0.05	+	42.85	1.73E-03	3.75E-02	2.080278	13.22981
response to antibiotic (GO:0046677)	79	5	2	0.05	+	42.85	1.73E-03	3.80E-02	2.074855	13.19532
smooth muscle contraction (GO:0006939)	80	5	2	0.05	+	42.85	1.73E-03	3.84E-02	2.070559	13.168
cellular carbohydrate metabolic process (GO:00442	81	23	3	0.21	+	13.97	1.73E-03	3.89E-02	2.065243	13.13419
cellular amino acid metabolic process (GO:0006520)	83	88	5	0.82	+	6.09	1.73E-03	3.93E-02	2.061032	13.10741
response to organic substance (GO:0010033)	84	285	9	2.66	+	3.38	1.72E-03	3.96E-02	2.057897	13.0994
homeostatic process (GO:0042592)	82	346	10	3.23	+	3.1	1.83E-03	3.92E-02	2.062081	12.9982

porphyrin-containing compound metabolic process	85	6	2	0.06	+	35.71	2.30E-03	4.80E-02	1.977368	12.01221
positive regulation of blood pressure (GO:0045777)	86	6	2	0.06	+	35.71	2.30E-03	4.85E-02	1.972961	11.98543

Supplementary table 5. Gene ontology terms of the biological process category associated with Taeeumin type.

PANTHER GO-Slim Biological Process	no	Homo sap	Client Tex	Client Tex	Client Tex	Client Tex	Client Tex	Client Text Box	z-score	combined score
response to chemical (GO:0042221)	1	576	22	4.17	+	5.28	3.40E-10	6.11E-07	4.987707439	108.7424
cholesterol homeostasis (GO:0042632)	2	14	6	0.1	+	59.2	4.45E-09	4.00E-06	4.611382362	88.67855
lipid metabolic process (GO:0006629)	3	120	10	0.87	+	11.51	3.48E-08	2.08E-05	4.256125479	73.0932
lipid homeostasis (GO:0055088)	4	33	6	0.24	+	25.11	3.35E-07	1.50E-04	3.791069449	56.52157
response to stimulus (GO:0050896)	6	1190	26	8.61	+	3.02	5.44E-07	1.95E-04	3.725407436	53.73646
drug metabolic process (GO:0017144)	5	127	9	0.92	+	9.79	6.21E-07	1.86E-04	3.737309385	53.41338
response to xenobiotic stimulus (GO:0009410)	7	40	6	0.29	+	20.72	9.23E-07	2.37E-04	3.675913309	51.07916
steroid metabolic process (GO:0008202)	8	44	6	0.32	+	18.84	1.53E-06	3.43E-04	3.580452268	47.94313
cellular catabolic process (GO:0044248)	9	111	8	0.8	+	9.96	2.37E-06	4.72E-04	3.496163158	45.28447
response to drug (GO:0042493)	10	81	7	0.59	+	11.94	3.36E-06	6.04E-04	3.429811812	43.22787
cellular metabolic process (GO:0044237)	11	1744	31	12.63	+	2.46	4.48E-06	6.70E-04	3.401569867	41.89335
cholesterol transport (GO:0030301)	12	12	4	0.09	+	46.04	4.36E-06	7.13E-04	3.384530874	41.77539
respiratory electron transport chain (GO:002290	13	57	6	0.41	+	14.54	6.05E-06	8.36E-04	3.340591926	40.13872
response to organic substance (GO:0010033)	14	285	11	2.06	+	5.33	1.01E-05	1.30E-03	3.215979761	36.99334
drug transport (GO:0015893)	15	38	5	0.28	+	18.18	1.39E-05	1.66E-03	3.145152983	35.1742
organic substance transport (GO:0071702)	16	112	7	0.81	+	8.63	2.47E-05	2.77E-03	2.992172165	31.74308
organic hydroxy compound metabolic process ((	18	46	5	0.33	+	15.01	3.23E-05	3.41E-03	2.928136777	30.27823
oxidation-reduction process (GO:0055114)	17	118	7	0.85	+	8.19	3.39E-05	3.38E-03	2.930883051	30.16493
energy derivation by oxidation of organic comp	19	100	6	0.72	+	8.29	1.18E-04	1.06E-02	2.555616077	23.1151
cellular respiration (GO:0045333)	20	100	6	0.72	+	8.29	1.18E-04	1.11E-02	2.539534806	22.96965
Unclassified (UNCLASSIFIED)	22	10588	53	76.65	-	0.69	1.62E-04	1.39E-02	2.459837335	21.46925
sterol metabolic process (GO:0016125)	21	35	4	0.25	+	15.79	1.73E-04	1.35E-02	2.470299428	21.39827
cellular response to chemical stimulus (GO:0070	23	208	8	1.51	+	5.31	1.73E-04	1.41E-02	2.454705623	21.2632
response to cytokine (GO:0034097)	28	118	6	0.85	+	7.02	2.78E-04	2.08E-02	2.311595226	18.92709
purine nucleobase transport (GO:0006863)	25	2	2	0.01	+	> 100	3.05E-04	1.96E-02	2.333918306	18.89353
cellular aromatic compound metabolic process (	24	77	5	0.56	+	8.97	3.14E-04	1.94E-02	2.337754253	18.8566
nucleobase transport (GO:0015851)	27	2	2	0.01	+	> 100	3.05E-04	2.03E-02	2.320756305	18.78698
nucleoside transport (GO:0015858)	29	2	2	0.01	+	> 100	3.05E-04	2.11E-02	2.306190324	18.66907
response to oxygen-containing compound (GO:1	26	78	5	0.56	+	8.85	3.32E-04	1.99E-02	2.328228003	18.64998
pyrimidine nucleoside transport (GO:0015864)	30	2	2	0.01	+	> 100	3.05E-04	2.19E-02	2.292097806	18.55499
cholesterol metabolic process (GO:0008203)	31	19	3	0.14	+	21.81	5.07E-04	2.85E-02	2.190334314	16.61807
cell-cell signaling (GO:0007267)	32	523	12	3.79	+	3.17	5.01E-04	2.90E-02	2.183486528	16.59211
cell communication (GO:0007154)	33	527	12	3.82	+	3.15	5.35E-04	2.91E-02	2.182129182	16.43851
apoptotic process (GO:0006915)	34	321	9	2.32	+	3.87	6.55E-04	3.46E-02	2.113008972	15.49021
programmed cell death (GO:0012501)	36	327	9	2.37	+	3.8	7.45E-04	3.82E-02	2.072702231	14.92786
synaptic signaling (GO:0099536)	35	331	9	2.4	+	3.76	8.10E-04	3.73E-02	2.082465182	14.82398
anterograde trans-synaptic signaling (GO:00989	38	330	9	2.39	+	3.77	7.93E-04	3.85E-02	2.069491374	14.77552
trans-synaptic signaling (GO:0099537)	37	331	9	2.4	+	3.76	8.10E-04	3.83E-02	2.071629571	14.74685
chemical synaptic transmission (GO:0007268)	39	330	9	2.39	+	3.77	7.93E-04	3.96E-02	2.057897227	14.69274
cytokine-mediated signaling pathway (GO:00192	40	98	5	0.71	+	7.05	8.93E-04	4.01E-02	2.052717333	14.41197
cellular response to cytokine stimulus (GO:0071	41	100	5	0.72	+	6.91	9.73E-04	4.26E-02	2.027610662	14.06174

cell death (GO:0008219)	43	342	9	2.48	+	3.64	1.01E-03	4.33E-02	2.020804566	13.93912
leukocyte chemotaxis (GO:0030595)	42	58	4	0.42	+	9.53	1.04E-03	4.32E-02	2.021771146	13.8866

Supplementary table 6. Gene ontology terms of the biological process category associated with Taeyangin type

PANTHER GO-Slim Biological Process	no	Homo sap	Client Tex	Client Tex	Client Tex	Client Tex	Client Tex	Client Tex	z-score	combined score
inorganic anion transport (GO:0015698)	1	44	9	0.19	+	47.72	1.19E-12	2.13E-09	5.987569	164.4011
mitochondrial electron transport, cytochrome c to oxygen (GO:000	2	185	13	0.79	+	16.39	2.59E-12	2.33E-09	5.972951	159.3545
nitric oxide biosynthetic process (GO:0006809)	4	54	9	0.23	+	38.88	6.12E-12	3.66E-09	5.89887	152.3056
sensory perception of sweet taste (GO:0050916)	3	55	9	0.24	+	38.17	7.09E-12	3.18E-09	5.922026	152.0322
arginine catabolic process (GO:0006527)	7	66	9	0.28	+	31.81	3.11E-11	1.12E-08	5.711477	138.1824
negative regulation of blood pressure (GO:0045776)	5	10	6	0.04	+	100	3.95E-11	1.01E-08	5.729041	137.2376
regulation of lyase activity (GO:0051339)	6	42	8	0.18	+	44.44	3.72E-11	1.11E-08	5.713003	137.1961
positive regulation of phosphate metabolic process (GO:0045937)	8	45	8	0.19	+	41.47	6.07E-11	1.21E-08	5.698311	134.0532
secondary metabolite biosynthetic process (GO:0044550)	9	45	8	0.19	+	41.47	6.07E-11	1.36E-08	5.67835	133.5836
ATP synthesis coupled electron transport (GO:0042773)	10	4072	45	17.45	+	2.58	8.22E-11	1.48E-08	5.663866	131.5255
mitochondrial ATP synthesis coupled electron transport (GO:00427	11	50	8	0.21	+	37.33	1.29E-10	2.11E-08	5.602731	127.581
ATP metabolic process (GO:0046034)	12	29	7	0.12	+	56.31	1.54E-10	2.30E-08	5.587773	126.2505
oxidative phosphorylation (GO:0006119)	13	55	8	0.24	+	33.93	2.56E-10	3.29E-08	5.525259	122.03
glucose homeostasis (GO:0042593)	14	55	8	0.24	+	33.93	2.56E-10	3.54E-08	5.512387	121.7457
ATP synthesis coupled proton transport (GO:0015986)	15	90	9	0.39	+	23.33	3.95E-10	4.73E-08	5.461173	118.246
energy coupled proton transport, down electrochemical gradient (	16	67	8	0.29	+	27.86	1.07E-09	1.20E-07	5.293496	109.3404
pyruvate metabolic process (GO:0006090)	17	73	8	0.31	+	25.57	2.00E-09	2.11E-07	5.189376	103.9438
lipid catabolic process (GO:0016042)	20	84	8	0.36	+	22.22	5.57E-09	5.56E-07	5.005906	95.1416
aromatic compound biosynthetic process (GO:0019438)	19	85	8	0.36	+	21.96	6.08E-09	5.46E-07	5.0094	94.76914
purine ribonucleotide metabolic process (GO:0009150)	18	28	6	0.12	+	49.99	6.24E-09	5.33E-07	5.014036	94.7266
ribonucleotide metabolic process (GO:0009259)	21	85	8	0.36	+	21.96	6.08E-09	5.74E-07	4.999766	94.58687
monocarboxylic acid metabolic process (GO:0032787)	22	126	8	0.54	+	14.81	1.08E-07	8.80E-06	4.444736	71.2986
carboxylic acid metabolic process (GO:0019752)	23	186	9	0.8	+	11.29	1.52E-07	1.19E-05	4.379412	68.75408
proton transmembrane transport (GO:1902600)	24	157	8	0.67	+	11.89	5.33E-07	3.99E-05	4.108058	59.33985
ATP biosynthetic process (GO:0006754)	25	547	13	2.34	+	5.54	7.21E-07	5.18E-05	4.047353	57.24021
cellular chemical homeostasis (GO:0055082)	26	117	7	0.5	+	13.96	1.02E-06	7.06E-05	3.974255	54.82766
ribose phosphate metabolic process (GO:0019693)	27	40	5	0.17	+	29.16	1.35E-06	8.99E-05	3.916349	52.93105
response to xenobiotic stimulus (GO:0009410)	28	3338	33	14.31	+	2.31	1.64E-06	1.05E-04	3.878737	51.66794
purine nucleoside triphosphate biosynthetic process (GO:0009145)	29	49	5	0.21	+	23.8	3.40E-06	2.10E-04	3.706673	46.67344
nucleoside triphosphate catabolic process (GO:0009143)	30	282	9	1.21	+	7.45	4.26E-06	2.55E-04	3.657185	45.22564
glycolipid metabolic process (GO:0006664)	31	23	4	0.1	+	40.57	5.05E-06	2.93E-04	3.621412	44.16718
oxoacid metabolic process (GO:0043436)	33	55	5	0.24	+	21.21	5.75E-06	3.23E-04	3.596119	43.39189
purine ribonucleoside triphosphate biosynthetic process (GO:0009	32	100	6	0.43	+	14	6.19E-06	3.17E-04	3.600996	43.18522
regulation of blood pressure (GO:0008217)	34	100	6	0.43	+	14	6.19E-06	3.27E-04	3.592914	43.0883
positive regulation of cellular biosynthetic process (GO:0031328)	35	221	8	0.95	+	8.44	6.18E-06	3.36E-04	3.585836	43.00921
tricarboxylic acid cycle (GO:0006099)	36	1744	21	7.48	+	2.81	1.28E-05	6.40E-04	3.414071	38.46314
organic acid metabolic process (GO:0006082)	37	118	6	0.51	+	11.86	1.52E-05	7.39E-04	3.374686	37.43949
cyclic nucleotide metabolic process (GO:0009187)	38	76	5	0.33	+	15.35	2.51E-05	1.19E-03	3.241266	34.33357
positive regulation of nucleobase-containing compound metabolic	39	208	7	0.89	+	7.85	3.79E-05	1.75E-03	3.129675	31.86184
coenzyme biosynthetic process (GO:0009108)	40	45	4	0.19	+	20.74	5.68E-05	2.55E-03	3.017344	29.49748
heterocycle biosynthetic process (GO:0018130)	41	16	3	0.07	+	43.74	6.93E-05	3.04E-03	2.963664	28.3832

biomineral tissue development (GO:0031214)	42	159	6	0.68	+	8.8	7.58E-05	3.24E-03	2.944	27.93094
sulfur compound transport (GO:0072348)	43	265	7	1.14	+	6.16	1.65E-04	6.90E-03	2.701632	23.53004
monosaccharide metabolic process (GO:0005996)	44	62	4	0.27	+	15.05	1.83E-04	7.46E-03	2.67558	23.02611
nucleoside triphosphate biosynthetic process (GO:0009142)	45	120	5	0.51	+	9.72	1.99E-04	7.75E-03	2.662772	22.69269
generation of precursor metabolites and energy (GO:0006091)	46	576	10	2.47	+	4.05	1.98E-04	7.90E-03	2.656314	22.65104
inorganic cation transmembrane transport (GO:0098662)	47	26	3	0.11	+	26.92	2.54E-04	9.69E-03	2.5867	21.41316
ion transmembrane transport (GO:0034220)	48	5	2	0.02	+	93.32	3.73E-04	1.34E-02	2.472958	19.52136
inorganic ion transmembrane transport (GO:0098660)	49	5	2	0.02	+	93.32	3.73E-04	1.37E-02	2.465035	19.45882
nucleoside triphosphate metabolic process (GO:0009141)	50	5	2	0.02	+	93.32	3.73E-04	1.40E-02	2.457263	19.39747
cofactor biosynthetic process (GO:0051188)	51	32	3	0.14	+	21.87	4.46E-04	1.57E-02	2.415816	18.63848
cellular nitrogen compound biosynthetic process (GO:0044271)	52	6	2	0.03	+	77.76	4.96E-04	1.71E-02	2.384551	18.14389
cellular homeostasis (GO:0019725)	53	34	3	0.15	+	20.58	5.26E-04	1.78E-02	2.369752	17.89213
purine ribonucleotide biosynthetic process (GO:0009152)	54	35	3	0.15	+	20	5.69E-04	1.89E-02	2.347497	17.53963
ossification (GO:0001503)	56	7	2	0.03	+	66.65	6.36E-04	2.08E-02	2.311595	17.01406
protein kinase B signaling (GO:0043491)	55	10588	29	45.39	-	0.64	6.47E-04	2.07E-02	2.313412	16.98776
nucleotide phosphorylation (GO:0046939)	57	39	3	0.17	+	17.95	7.65E-04	2.37E-02	2.261958	16.23098
regulation of G1/S transition of mitotic cell cycle (GO:2000045)	58	39	3	0.17	+	17.95	7.65E-04	2.41E-02	2.255531	16.18487
aerobic respiration (GO:0009060)	59	44	3	0.19	+	15.91	1.06E-03	3.24E-02	2.139441	14.65407
ribonucleotide biosynthetic process (GO:0009260)	60	12	2	0.05	+	38.88	1.59E-03	4.75E-02	1.981815	12.77086
